# Societies of Futures Past: Examining the History and Potential of International Society Collaborations in Addressing the Burden of Rheumatic Heart Disease in the Developing World

**DOI:** 10.3389/fcvm.2021.740745

**Published:** 2021-11-02

**Authors:** Zachary Obinna Enumah, Percy Boateng, Ralph Morton Bolman, Friedhelm Beyersdorf, Liesl Zühlke, Maurice Musoni, Adriano Tivane, Peter Zilla

**Affiliations:** ^1^Cardiac Surgery Intersociety Alliance, Cape Town, South Africa; ^2^University of Minnesota, Minneapolis, MN, United States; ^3^Department of Cardiovascular Surgery, University Hospital Freiburg, Freiburg im Breisgau, Germany; ^4^Faculty of Medicine, Albert-Ludwigs-University Freiburg, Freiburg im Breisgau, Germany; ^5^Division of Paediatric Cardiology, Department of Paediatrics and Child Health, Red Cross War Memorial Children's Hospital, University of Cape Town, Cape Town, South Africa; ^6^Division of Cardiology, Department of Medicine, Groote Schuur Hospital, University of Cape Town, Cape Town, South Africa; ^7^Cape Heart Institute (CHI), Faculty of Health Sciences, University of Cape Town, Cape Town, South Africa; ^8^Faculty of Health Sciences, Institute of Infectious Disease and Molecular Medicine (IDM), University of Cape Town, Cape Town, South Africa; ^9^King Faisal Hospital Kigali, Kigali, Rwanda; ^10^Hospital Central Maputo, Maputo, Mozambique

**Keywords:** cardiac surgery, academic societies, rheumatic heart disease, registry, cardiac surgery intersociety alliance, CSIA

## Abstract

This paper explores the role and place of national, regional, and international society collaborations in addressing the major global burden of rheumatic heart disease (RHD). On the same order of HIV, RHD affects over 40 million people worldwide. In this article, we will outline the background and current therapeutic landscape for cardiac surgery in low- and middle-income countries (LMICs) including the resource-constrained settings within which RHD surgery often occurs. This creates numerous challenges to delivering adequate surgical care and post-operative management for RHD patients, and thus provides some context for a growing movement for and applicability of structural heart approaches, innovative valve replacement technologies, and minimally invasive techniques in this setting. Intertwined and building from this context will be the remainder of the paper which elaborates how national, regional, and international societies have collaborated to address rheumatic heart disease in the past (e.g., Drakensberg Declaration, World Heart Federation Working Group on RHD) with a focus on primary and secondary prevention. We then provide the recent history and context of the growing movement for how surgery has become front and center in the discussion of addressing RHD through the passing of the Cape Town Declaration.

## Introduction

Rheumatic heart disease (RHD) affects up to 40 million individuals globally (1). Caused by sequalae of skin or throat infection from streptococcal infection, RHD is endemic in low- and middle-income countries, and it is the most common cardiovascular pathology in young individuals aged 25 and younger (2, 3). Standing as a stark challenge to the international cardiac surgery community, the unmet need for cardiac surgery for rheumatic heart disease is estimated to be 300 to 400 operations per million population in low-income countries (4, 5). Recent global attention has addressed the burden of RHD with commitments to strengthening preventative efforts, as well as surgical efforts, with the adoption of the Drakensberg Declaration in 2006, the World Health Organization Resolution against rheumatic fever (RF) and RHD in 2018 and Cape Town Declaration on Access to Cardiac Surgery in the Developing World in 2018, respectively.

These efforts are commendable and will be elaborated upon further in this article. The current therapeutic landscape for addressing RHD is multidimensional and has many challenges. We will first provide a review of the therapeutic ecology of RHD treatment, specifically focusing on the current state of affairs of cardiac surgery for RHD in LMICs (6). This discussion will focus on the epidemiology of surgery for RHD, current challenges, and ongoing efforts to address these challenges, including an emerging environment for interventional techniques in resource poor settings (7–9). We will then focus on the history and roles of international medical and surgical societies, as well as global health agencies, in addressing the burden of RHD. Finally, and in the context of multisectoral collaboration, we will comment on the need and role of research and the lack of a robust, cardiac surgical registry for RHD patients (10).

### Therapeutic Landscape of Surgical Treatment for RHD

Surgery is a cornerstone of any health care system. Nevertheless, it has been estimated that worldwide 5 billion people lack access to safe or affordable surgery and anesthesia (11). For cardiac surgery specifically, it is estimated that up to 6 billion people have inadequate, or insufficient access to cardiac surgery, if they have any access at all (12). Despite cardiovascular disease being the leading cause of death worldwide, enormous disparities exist between high-income countries and LMICs. Referral for adequate care and completion of care (e.g., surgery) is a major problem along the spectrum of management for RHD. For example, in the VALVAFRIC study by Kingue et al. (13), 1,200 patients were determined to be in need of surgery, yet only 27 patients (2.2%) were able to receive it. Even for those patients who can receive surgery, there are other challenges.

Mortality due to RHD is high in LMICs, especially among young individuals. For example, a recent study by Okello et. al. (14) reported a mortality rate of over 17% with a median age < 30. Similarly, Gunther and colleagues have suggested up to a 12.5% mortality rate in Ethiopia (15). Post-operative care is a major challenge in the surgical management of RHD. Complications from surgery may be plentiful, such as stroke or bleeding. Some data suggest bleeding or thrombotic events may be low (16). Others, though, suggest that complications from thrombosis may be quite significant. For example, a recent study by Scherman et al. (17) showed that almost every fourth patient needing aortic valve replacement for RHD in a middle-income country like South Africa either had a stroke or had died of valve related complications 10 years after aortic valve replacement with a mechanical prosthesis. It is important to note that, in the context of access to cardiac surgery, in many resource-poor countries, reoperations are simply not offered, and some outcomes cannot be assessed without standard follow-up or mechanisms to capture patient outcomes.

In order for more patients to be able to receive treatment and to improve the safety of post-operative care for patients receiving surgery, the majority of these patients urgently require two major developments in replacement valves to address their needs. The first, and perhaps most pressing requirement is for improved valve design, incorporating long-lasting valve leaflet materials that will allow implantation into young patients without the need for anticoagulation. The other area in need of attention is to develop and implement simplified and reproducible transcatheter procedures that can deploy such valves cost-effectively, thereby significantly increasing the capacity of low-volume hospitals (7). A combination of easy-to-place trans-catheter technologies tailor-made for non-sophisticated medical facilities and suitable for the often compliant, non-calcified valves of patients with RHD, constructed with long-lasting leaflet material, promises to eventually introduce replacement valves that specifically address the “needs of the many” (8, 9). At the same time, advances in strengthening health systems to provide more access to sustainable cardiac surgical care is needed, as that will provide an avenue for valve repair in addition to minimally invasive valve replacement. Equally as important, a stronger health system will also provide avenues for improved medical management of heart failure for these vulnerable patients both pre and post-intervention.

There are many challenges to delivering sustainable cardiac surgical care in LMICs. On a broad scale, these include lack of health care infrastructure, critical gaps in the health care workforce, lack of training opportunities for critical providers, insufficient funding at the government level, competing health care priorities, and poor tracking of outcomes (18). Specifically, there is a lack of availability of multidisciplinary training including for perfusionists, anesthesiologists, and nurses, as well as surgeons and cardiologists. Additionally, the cost and labor intensive aspects of cardiac surgery pose substantial barriers, especially in the context of resource-poor countries having to consider and juxtapose health efforts to serve large populations (19). Research has been done on understanding the various cost models for cardiac surgery in resource-poor settings (20, 21) with estimates of approximately $6,000–11,000 per patient (22). Despite open heart surgery in a low- and middle-income context costing approximately $10,000 per case (including the cost of cardiopulmonary bypass circuit and oxygenator, heart valves, intensive care and hospital associated costs, as well as other consumables such as drugs, blood gases, and other laboratory tests), cardiac surgery is often relegated to the back burner. There is a growing body of evidence that cardiac surgery may be more cost effective than previously understood (23).

Other challenges appear on a more local level in terms of advancing the cardiac surgical agenda. For example, there are few regional centers of excellence for cardiac surgery in sub-Saharan Africa. While centers throughout the continent do perform cardiac surgery, few pathways exist for collaboration. Furthermore, training of key providers, and the volume to support such training is still lacking (4).

### The History of Attempts by Societies, Collaborations and Conferences to Address RHD

The concept of a role for international agencies and academic societies in collaborating to address capacity for cardiac surgery in the developing world is, in and of itself, a relatively recent initiative. Societies serve as an integral part of the academic and service community in terms of addressing and promoting clinical care, research, and education in cardiac surgery. In this section, we will provide a brief history of recent conferences with resultant publications and importantly policy documents which have been integral to the further advancement of the agenda to combat RHD.

In October 2005, delegates to the 1st All Africa Workshop on Rheumatic Fever and Rheumatic Heart Disease gathered in the Drakensberg mountains, South Africa and called out and acknowledged the large burden of RHD on the continent with an appreciation for the sociopolitical nature of its endemicity. The result was what has come to be known as the Drakensberg Declaration, with authors representing institutions from across the continent and the world including individuals representing the University of Cape Town, New York Medical College, University of Ibadan, University of Ghana Medical School, University of Melbourne, World Health Organization, University of Zimbabwe, Harare, University of Nairobi and the University of Libreville, to name a few (24). The result of this declaration was supporting the development of 1) public health awareness (both the lay public and health care workers) of RHD; 2) improving information quality vis-à-vis epidemiological surveillance; 3) advocacy; and 4) establishing national primary and secondary prevention programs for RHD (25). The underlying concept was a “bottoms up approach” with the central tenets being Awareness, Surveillance, Advocacy and Prevention, known as A.S.A.P and subsequently adopted across Africa.

In 2013, the World Heart Federation called for a reduction in premature RHD mortality by 25% by 2025 and the World Congress of Paediatric Cardiology and Cardiac Surgery brought together over 300 delegates at the 2nd RHD forum to discuss needs, opportunities and research in RHD, including the need for access to surgery in LMICs (26–28). One year later, in February 2014, the Second Rheumatic Fever/Rheumatic Heart Disease Workshop convened over 80 delegates in Zambia, from which emerged the Mosi-o-Tunya Call to Action. Again, representing institutions from 13 African countries and others, the message of this Mosi-o-Tunya Call to Action was to call for the elimination of RF and control of RHD in Africa in our lifetime (29). The Third All Africa Workshop on Rheumatic Fever and RHD occurred in February 2015. It was hosted by the Social Cluster of the African Union Commission and convened RHD experts in Addis Ababa, Ethiopia. These experts were both clinicians and researchers affiliated with the Pan-African Society of Cardiology (PASCAR) and also included representation from countries across the continent and the world. There were seven key recommendations from the Addis Ababa Communique: 1) establish prospective RHD registries; 2) ensure supplies of penicillin to promote primary and secondary prevention; 3) ensure access to reproductive health for women with RHD; 4) decentralize diagnostic capabilities to district hospitals including access to point-of-care technologies; 5) institute cardiac surgery centers of excellence for both sustainable care delivery and training; 6) promote national RHD control programs; and 7) foster partnerships to carry out the above recommendations (30). Other important events included the passing of the Cairo Accord (31, 32), the Khartoum Action Plan (33): culminating in a major milestone in May 2018 when all member states of the World Health Organization unanimously agreed to, and adopted, the Resolution on Rheumatic Fever and Rheumatic Heart Disease at the 71st World Health Assembly (34).

Local societies in endemic countries have played an important role in maintaining momentum, promoting partnerships, and outlining a clear and detailed path forward for curbing the burden of RHD e.g., Algerian Society of Cardiology. And, in the period between 1990 and 2015, deaths, disability-adjusted life years (DALYs) and years of life lost have all decreased. All this notwithstanding, however, new estimates of the prevalence of RHD are as high as 40 million, with stabilization of the mortality estimates in the 2019 Global burden of disease reports and it is estimated that as many as 300–400 operations per million population surgical procedures are needed for RHD patients in certain LMIC settings (4, 35). In light of this continued burden of disease, and especially the surgical aspects in addressing it, a major contribution on the international stage for RHD was the Cape Town Declaration on Access to Cardiac Surgery in the Developing World, launched at the 97th conference of the American Association of Thoracic Surgery in Boston (May 2017) and signed at the 50th anniversary of the 1st heart transplant at Groote Schuur Hospital, University of Cape Town.

### Surgical Societies in Addressing the Burden of RHD

Convened by Professor Peter Zilla, the then-Chair of the Christiaan Barnard Division of Cardiothoracic Surgery, Faculty of Health Sciences, University of Cape Town, Cape Town, South Africa, delegates and signatories of the Cape Town Declaration represented cardiothoracic/cardiovascular societies from across the globe, such as the Society of Thoracic Surgeons, American Association for Thoracic Surgery, Australian and New Zealand Society of Cardiac and Thoracic Surgeons, Society of Cardiothoracic Surgeons of South Africa, Pan-African Society of Cardiology, Brazilian Society of Cardiovascular Surgery, European Association for Cardio-Thoracic Surgery, World Heart Federation, Asian Society for Cardiovascular and Thoracic Surgery, Chinese Society for Thoracic and Cardiovascular Surgery, Pan-African Society for Cardiothoracic Surgery, and the South African Heart Association. Additionally, delegates represented many humanitarian organizations, governments, industry and academia (36).

The overarching theme of the Cape Town Declaration is unique to the previous multi-sectorial and international declarations and communiques. For perhaps the first time in modern history, surgery was front and center. The mission of the Cape Town Declaration was “to urge all relevant entities within the international cardiac surgery, industry and government sectors to commit to develop and implement an effective strategy to address the scourge of RHD in the developing world through increased access to life-saving cardiac surgery” (36). This was undertaken by two main aims including the formation of an international working group dedicated to this cause (henceforth known as the Cardiac Surgery Intersociety Alliance, CSIA) and the investment in training of cardiac surgeons and other important staff in LMICs. The announcement of the Cape Town Declaration was published in multiple (9) cardiovascular surgery journals simultaneously, signaling the commitment of the global cardiac surgery community to redoubling their efforts to reduce the burden of RHD.

The CSIA recently announced its endorsement of two pilot sites in Rwanda and Mozambique, including King Faisal Hospital Kigali (Rwanda) and Hospital Central Maputo (Mozambique) (37). International societies and conferences have maintained and increased the momentum necessary to address the burden of RHD. In important ways, the collaboration of these international societies holds forth the promise of finally addressing this formerly intractable problem of providing access to cardiac surgery for the countless millions who until now have been deprived of its benefits. Importantly, societies, aided and coordinated by the CSIA, may play an important role in furthering and facilitating regional collaborations between sites. For example, preliminary discussions between the cardiac surgery teams at King Faisal Hospital Kigali and Hospital Central Maputo have been facilitated, and planning is underway for regional cooperation between the two centers where staff may visit one another for mutual benefit. The CSIA will provide sponsorship for these trips.

Additionally, groups such as the CSIA can leverage and build upon previously existing efforts and strong networks of collaboration that have manifested from the All-Africa Workshops on Rheumatic Fever and RHD. The CSIA plans to partner and build on ongoing efforts in the region to build a robust, prospective, multicenter registry for RHD surgical patients, including following them in the post-operative period. Previous registry work, such as the REMEDY and VALVAFRIC, have made important contributions to our understanding of RHD in LMICs (13, 38). Smaller registries, such as the work of Ntaganda and colleagues, are laying important groundwork for future, robust, multi-center registries that the CSIA hopes to support (39). Again, surgical societies can and are playing a major role in addressing the massive global burden of RHD.

## Conclusion

As we look to the future of cardiac surgery for RHD, it is clear it will continue to require a multisectoral and multidisciplinary effort. As part of that effort, we should not underestimate the role that collaborations between international societies, agencies, and other involved parties can play in furthering the agenda to reduce the enormous burden of RHD. Recent history has demonstrated that some trends for RHD mortality and morbidity have moved in the right direction, and international collaborations may have played a major role in achieving that. Nevertheless, the surgical burden of RHD remains high, and heretofore has been largely unaddressed. For many patients with symptomatic RHD and major valvular pathology, surgery remains the only effective treatment. Manifest through these new collaborations, we anticipate that cardiac surgical societies have a crucial role in ensuring increased access to life-saving cardiac surgery for RHD in the developing world. In doing so, we will begin to realize the vision of the Cape Town Declaration, the Addis Ababa Communique, and the many other declarations and hopes that have been laid bare to address and curb the burden of RHD for the most vulnerable populations.

## Data Availability Statement

The original contributions presented in the study are included in the article/supplementary material, further inquiries can be directed to the corresponding authors.

## Author Contributions

All authors listed have made a substantial, direct and intellectual contribution to the work, and approved it for publication.

## Conflict of Interest

The authors declare that the research was conducted in the absence of any commercial or financial relationships that could be construed as a potential conflict of interest.

## Publisher's Note

All claims expressed in this article are solely those of the authors and do not necessarily represent those of their affiliated organizations, or those of the publisher, the editors and the reviewers. Any product that may be evaluated in this article, or claim that may be made by its manufacturer, is not guaranteed or endorsed by the publisher.
